# Effects of Different Surface Native Pre-Oxides on the Hot Corrosion Properties of Nickel-Based Single Crystal Superalloys

**DOI:** 10.3390/ma13245774

**Published:** 2020-12-17

**Authors:** Zehao Chen, Shusuo Li, Mengmeng Wu, Yanling Pei, Shengkai Gong, Heng Zhang

**Affiliations:** School of Material Science and Engineering, Beihang University, Xueyuan Road, Beijing 100191, China; buaachenzehao@163.com (Z.C.); lishs@buaa.edu.cn (S.L.); wmm_121@126.com (M.W.); peiyanling@buaa.edu.cn (Y.P.); gongsk@buaa.edu.cn (S.G.)

**Keywords:** superalloys, surface native pre-oxides, oxidation, hot corrosion, sulfides

## Abstract

A study is carried out on the effect of different surface native pre-oxides on hot corrosion of single crystal nickel-based superalloy at 900 °C. The effect of different oxides formed by different superalloys through pre-oxidation on hot corrosion is verified by normal hot corrosion and tube sealing experiments. The relationship between different surface oxides and the effect of different surface oxides layer on the hot corrosion properties of alloys are studied. In summary, the stable and dense surface pre-Al_2_O_3_ layer which can be obtained by pre-oxidation has an obvious positive effect on the improvement of superalloy hot corrosion resistance in reaction. In addition, the internal sulfides are analyzed in depth, and the relationship between Cr, Mo, O and S is discussed in detail.

## 1. Introduction

The industry gas turbines (IGTs) have the highest thermal-power conversion efficiency and a relatively long service life, but their service life and strength are generally affected by the harsh environment [1]. Therefore, with the continuous development of gas turbine, there are more and higher requirements are put forward for the manufacture of superalloys for gas turbine correspondingly and Nickel-based single crystal superalloys become the preferred turbine blade materials for industrial gas turbine. Compared with the aero gas turbines that usually use clean fuels treated by desulfurization, industry gas turbines and marine gas turbine are more susceptible to hot corrosion due to the use of primitive fuel for reducing costs, the high oxygen concentration in the service environment and the high vulnerability to corrosion by seawater. In general, the combustion of the incomplete desulfurization industrial gas turbine fuel used in power plants promotes the production of “corrosive” sodium sulfate. In the meantime, water is used to obtain better heat dissipation and higher turbine efficiency, but the NaCl in non-pure water will bring bad effects in hot corrosion on the service process of superalloys [2,3].

Hot corrosion has a significant effect on the failure process of superalloys [4,5]. Especially, the hot corrosion occurred at 900 °C which is referred to as the Type-I hot corrosion [6]. It is important to note that the oxidation of single crystal superalloy at this temperature is usually not severe as hot corrosion. Thus, although hot corrosion is essentially an oxidation behavior, it is quite different from what is commonly referred to as “oxidation” of the alloy in direct reaction with oxygen in the air [7]. However, oxidation and hot corrosion often occur alternately as the change in temperature caused by a change in engine power and affect each other, so it is necessary to study the relationship between them [8]. To better distinguish between oxidation and hot corrosion, the fluxing model theory which explains that hot corrosion is based on the movement of oxygen ions and metal salts ions in the molten salt film formed at 800–900 °C as well as the loss and gain of electrons inside the materials are proposed and verified after a series of researches in order to explain the mechanism of hot corrosion [4,9]. A large number of researchers have used this model to analyze and simulate the experimental process, which can explain the accelerated reaction process caused by hot corrosion accurately, and why the experimental samples cannot produce stable oxide layers on the surface during hot corrosion [7,10,11,12]. However, these studies still have an obvious limitation which neglects the formation of different oxides simultaneously will lead to great difference in fluxing results as the complicated system of superalloys. Other researchers have chosen to treat the surface of the superalloys to produce a very dense protective oxide layer and then conduct hot corrosion tests [13,14,15,16,17]. This method can well elucidate the interaction between oxides and molten salt, but when hot corrosion occurs, it is difficult to form such a “perfect” oxide layer on the surface of the superalloy. In addition, some surface coatings are added to improve the oxidation and hot corrosion resistance of superalloys [18,19,20,21]. These coatings can effectively improve the oxidation or hot corrosion resistance of the alloys to some extent, but their composition is complex and the degradation of the coating can be caused by diffusion. Furthermore, achieving both simultaneously is difficult.

In this study, three kinds of single crystal superalloys with different Cr contents are used to systematically analyze the influence of pre-oxidation behaviors of the alloys on the hot corrosion and the distribution of internal sulfides. Through specialized experimental design, the enhancement effect of different surface pre-oxidation products on hot corrosion resistance and the relationship of the oxides are analyzed in detail. It is of great scientific and engineering significance to study the hot corrosion behavior of different alloys after pre-oxidation, which is helpful to understand the role of different oxides in hot corrosion, describe the hot corrosion behavior after oxidation and provide ideas for improving both hot corrosion and oxidation resistance of superalloys.

## 2. Experimental Procedures

### 2.1. Preparation of Materials

The nominal compositions of the three experimental alloys used are listed in Table 1.

**Figure 1:**
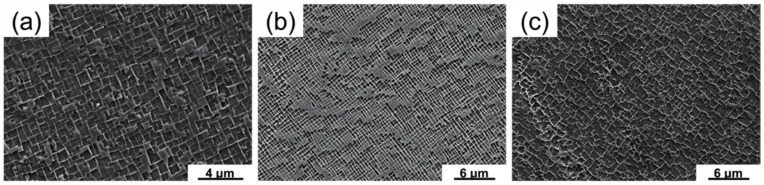
The microstructure morphologies of three nickel-based single crystal superalloys: (**a**) Alloy 1, (**b**) Alloy 2 and (**c**) Alloy 3.

The single crystal (SC) superalloy bars are directionally solidified by liquid metal cooling (LMC) method. The casting temperature is 1550 °C, the withdraw rate is 6 mm/min, the diameter is 10.5 mm, and the length is about 220 mm. The (001)—oriented Rene-n5 seed crystal is used to ensure the growth of rod crystal into single crystal. Then, the solution heat treatment of alloy 1 and alloy 2 is at 1310 °C for 2 h, 1315 °C for 2 h, 1320 °C for 4 h, and air cooling (AC); the solution heat treatment of alloy 3 is at 1305 °C for 2 h, 1310 °C for 8 h and air cooling (AC). After solution heat treatment, the aging heat treatment of 1120 °C/4 h/AC and 900 °C/12 h/AC is carried out for three alloys bars successively. Electro-spark wire-electrode cutting is used to cut the hot corrosion samples with 10 mm diameter and 3 mm thickness, and then cylindrical sides are machined by a lathe. The surfaces of all the three alloys specimens are ground by grinding to #1000 emery papers and cleaned with alcohol to remove dirt subsequently. The microstructure morphologies of three nickel-based single crystal superalloys are shown in Figure 1. The Figure 1a–c correspond to Alloy 1, Alloy 2 and Alloy 3 respectively.

### 2.2. Pre-Oxidation and Hot Corrosion Testing

To analyze the effect of the oxidation layer to the hot corrosion, a surface pre-oxidation experiment is carried out. Firstly, all samples receive a heat treatment at 1000 °C for 3 h (AC) and 1100 °C for 16 h (AC) which results in the samples quality remain unchanged to form a stable oxidation layer. Then, the hot corrosion tests are performed. In order to make the samples subjected to salt environment similar to the actual service of the material, the three kinds of specimens are sprayed with a saturated aqueous solution which is consist of 75 wt.% Na_2_SO_4_ + 25 wt.% NaCl and followed by drying. After that, each specimen is weighted to ensure that a 0.3–0.5 mg/cm^2^ salt is supplied on the surface of the sample [22,23]. Each sample is put into a crucible separately, and all crucibles are put into a box furnace at 900 °C for hot corrosion, and the kinetics of hot corrosion is studied. The experiment is suspended every 20 h to weigh the weight change with both “mass gain” (referring the total weight of sample and crucible, including the spallation from sample) and “sample weight change” (referring only the weight of the sample without crucible or spallation) and then salt is added to make sure enough salt for the subsequent reaction as is the case with gas turbines operating continuously in high salinity environments. Before each cycle of the weigh and salt replenishment, the samples surfaces are cleaned simply by sitting in deionized water for 5 min to ensure the accuracy of hot corrosion test to a certain extent [24]. When the experiment is over, specimens are taken out and the microstructures are observed. Meanwhile, the same set of tests are also conducted on alloys samples without the pre-oxidation treatment for comparison.

**Figure 2:**
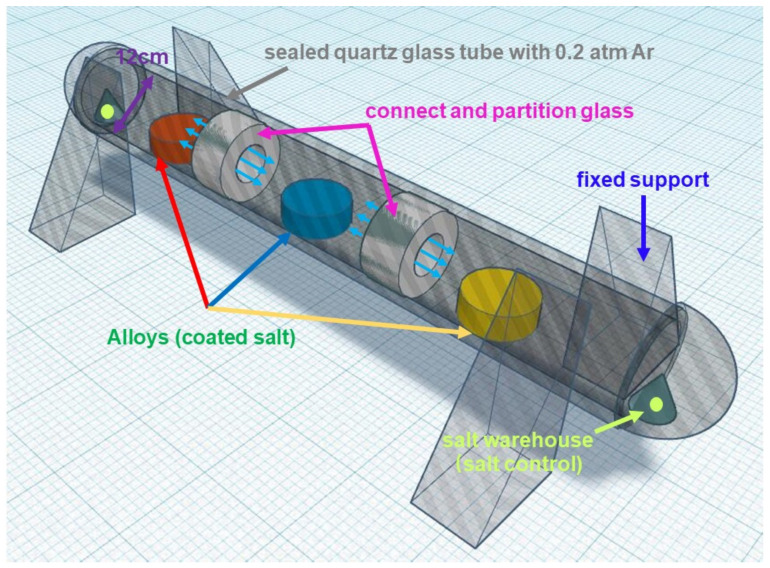
The design of tube sealing experiment.

A tube sealing method that have been improved before is also used as Figure 2 showed which can not only study the hot corrosion characteristics under low oxygen condition, but also reflect the actual atmosphere environment faced by the blade material when oxygen is kept at a low content due to altitude or combustion to some extent [25]. Three different Cr content samples after pre-oxidation are coated with saturated aqueous solution of salt (1.6–1.8 mg/cm^2^) to maintain the reaction operation within 200 h [26,27]. The three samples are sealed into one SiO_2_ glass tube with 0.2 atm high purity Ar to make sure the reaction of three specimens takes place under exactly the same circumstance. After 200 h, the samples are taken out and the microstructure evolution is observed.

### 2.3. Analyzing Methods

An analytical balance with 0.01 mg minimum sensitivity is used to prepare the salt solution weigh the mass change of the samples and weigh the mass change of the samples. X-ray diffraction (XRD) is used to identify the reaction products after oxidation and corrosion. Scanning electron microscopy (SEM) with SE (only surface structure) and BSE detectors is used to observe the surfaces and cross sections morphologies, energy dispersive spectroscopy (EDS) and electron backscattered diffraction (EBSD) are used to characterize the elements distribution and analyze the sulfides information.

## 3. Results

**Figure 3:**
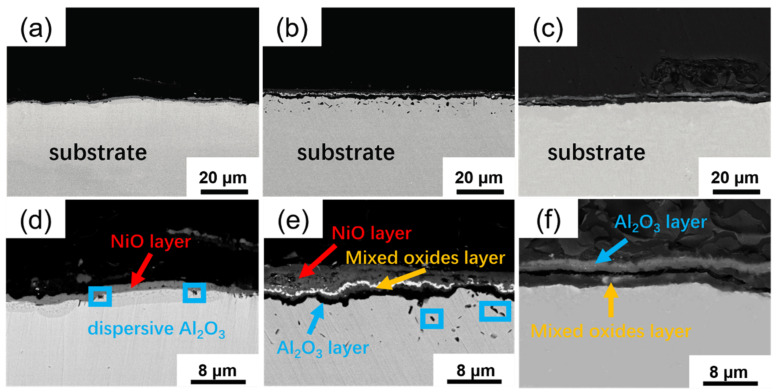
The cross-section structures (BSE images) of prefabricated oxidation layer for different alloys: (**a**,**d**) Alloy 1, (**b**,**e**) Alloy 2 and (**c**,**f**) Alloy 3.

The cross-section structures (BSE images) of prefabricated oxidation layer for different alloys are shown in Figure 3. It can be seen that all three alloys formed stable and different oxidation layers. Actually, the mass gain of three alloys started to remain constant when the end of the oxidation. It can be seen in Figure 3a,d that on the surface of Alloy 1, a continuous, steady NiO layer existed, a layer of solid solution oxides lay below, meanwhile, some dispersive Al_2_O_3_ distributed below the NiO layer. Figure 3b,e show that, for Alloy 2, the more complex oxidation layer appeared which was composed of outer loose oxide (NiO), continuous NiO/Ta_2_O_5_/Cr_2_O_3_ layer, thin Cr_2_O_3_ and Al_2_O_3_ mixed layer, continuous Al_2_O_3_ layer from outside in. Under the oxidation layer, there were some Al_2_O_3_ dispersive distributed in the substrate. Figure 3c,f indicate that a thin continuous outer Al_2_O_3_ layer and inner mixed oxides (Al_2_O_3_, Cr_2_O_3_ and NiO) layer formed in Alloy 3. The atomic percentages of elements at different locations measured by EDS in Figure 3 are listed in Table 2. Alloy 2 had the most continuous and complex multilayer oxide formed in the three alloys unquestionably.

**Figure 4:**
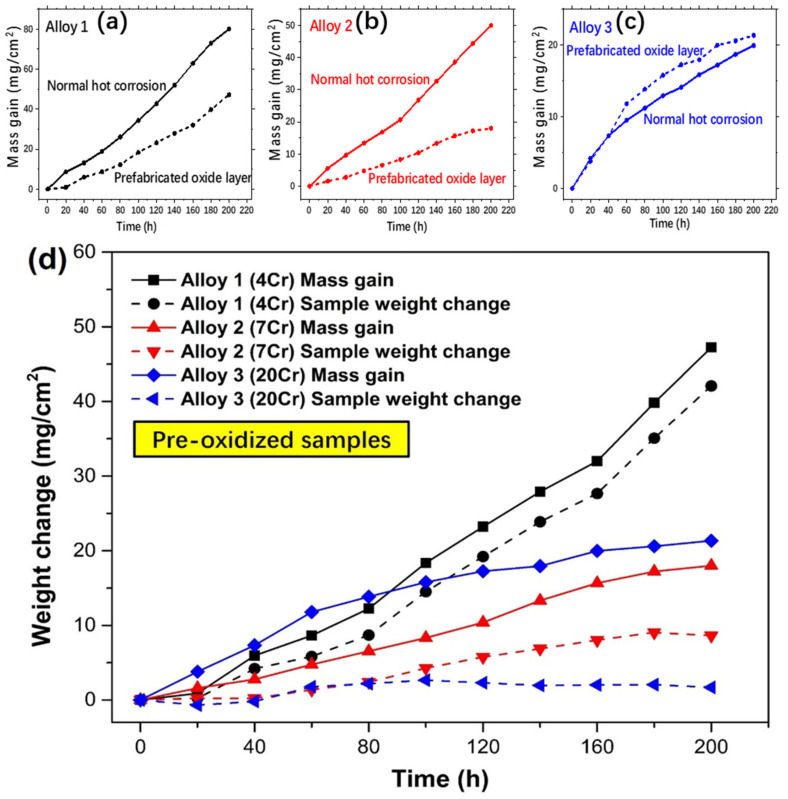
The hot-corroded kinetic curves of the prefabricated oxidation alloys compared to pure alloys in the atmosphere at 900 °C in 200 h: (**a**) Alloy 1, (**b**) Alloy 2, (**c**) Alloy 3 and (**d**) the weight changes of the three alloys specimens after hot corrosion.

The hot-corroded kinetics curves of the prefabricated oxidation alloys compared to pure alloys in the atmosphere at 900 °C in 200 h are illustrated in Figure 4. Figure 4a shows that the hot-corroded mass gain of Alloy 1 which had prefabricated oxide layer was five in eight of normal specimen after 200 h. The increasing rate of treated specimen curve kept stable and lower than normal specimen during the test. Figure 4b indicates that, for Alloy 2, the mass gain of treated specimen was just one third of normal corrosion sample. It also can be seen that the rate of mass rise began to slow down for treated specimen after 160 h while the normal hot corrosion specimen remained rapid mass gain. However, the distinction of mass gain between prefabricated oxide layer and normal hot corrosion was tiny for Alloy 3 as shown in Figure 4c. At first, the mass gain of normal test was a bit bigger than treated corrosion test. After 40 h, the mass of treated specimen surpassed normal specimen, but the gap had not kept widening. In order to compare the effect of pre-oxidation on hot corrosion resistance of alloys with different Cr content more directly, the mass gains (referring the total weight of sample and crucible, including the spallation from sample which can reflect the reaction degree of the specimens) and the sample weight changes (only the weight of the sample without crucible or spallation which can reflect the spallation degree of the reaction layer of the samples) of the three pre-oxidized alloys specimens during hot corrosion in the atmosphere were also shown in Figure 4d. Interestingly, Alloy 3 with high Cr content showed the largest increase in mass gain up to 80 h, although the distinction was not significant between 3 alloys. The mass gain growth rate of Alloy 1 kept increasing during hot corrosion test while the rates of Alloy 2 and 3 slowed down gradually. When the experiment was carried out to 200 h, the mass gain of Alloy 2 was still smaller compared with that of Alloy 3 (not big difference) and Alloy 1 showed the greatest mass gain. The sample weight change of Alloy 1 maintained the same increasing trend as that of the mass gain during the test. The case for Alloy 2 was similar, but not obvious because it changed little. For Alloy 3, the sample weight change remained basically unchanged since the beginning of the experiment. In addition to the above-mentioned cases, the gaps between the mass gain and the sample weight change of Alloy 1 and Alloy 2 (before 160 h) were not obvious during the reaction process, which may indicate that there is no obvious spallation. At the same time, although the gaps between mass gain and sample weight change of Alloy 2 (after 160 h) and Alloy 3 (after 60 h) increased slightly, they were also less than 20 mg/cm^2^ and were not sufficient to demonstrate the occurrence of severe spallation.

**Figure 5:**
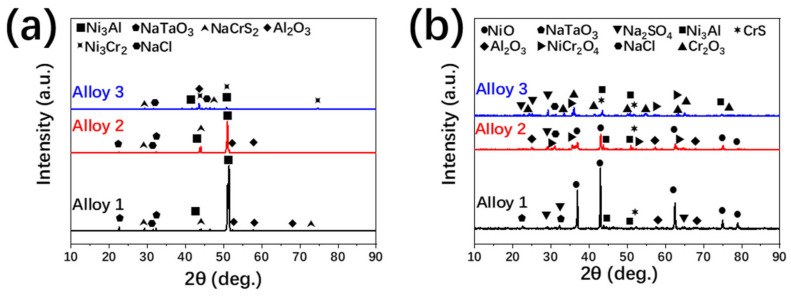
The XRD patterns of the corrosion products developed on the surface of the specimens after the corrosion experiments at 900 °C in 200 h for (**a**) tube-sealing and (**b**) no tube-sealing.

The XRD patterns of the corrosion products developed on the surface of the specimens after the corrosion experiments at 900 °C in 200 h for tube-sealing and no tube-sealing (both prefabricated oxidation layer) are shown in Figure 5. Figure 5a shows the products on the surface of different tube-sealing samples. There were NaTaO_3_, NaCrS_2_ and Al_2_O_3_ besides Ni_3_Al for Alloy1; the product variety of Alloy 2 was no difference compared to Alloy 1 but Ni_3_Al was less obvious while NaCrS_2_ was more remarkable; for Alloy 3, NaTaO_3_ disappeared, Ni_3_Cr_2_ formed and Ni_3_Al was further less obvious. The reaction products of the unsealed samples were more varied in comparison with tube-sealing samples as shown in Figure 5b. NiO was primary product on the surface of Alloy 1 after 200 h, and other products such as NaTaO_3_, CrS and Al_2_O_3_ also can be found. Besides the products that were indicated in Alloy 1, NiCr_2_O_4_ became an important component of surface formation in Alloy 2. NiO content decreased on the surface of the specimens in the meantime. Accompanied by Cr content increased in alloy, the major surface products changed to be Cr_2_O_3_ and CrS for Alloy 3. NiO and NaTaO_3_ cannot be found while NiCr_2_O_4_ was still existed.

**Figure 6:**
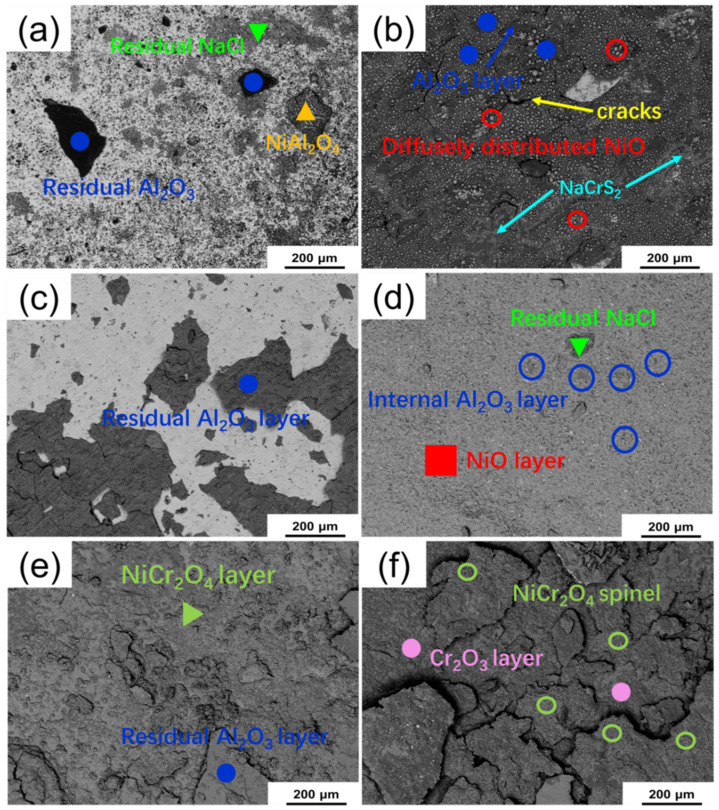
The surface morphologies (BSE images) of sealed and unsealed samples of three alloys corroded at 900 °C for 200 h: sealed samples: (**a**) Alloy 1, (**b**) Alloy 2 and (**c**) Alloy 3; unsealed samples: (**d**) Alloy 1, (**e**) Alloy 2 and (**f**) Alloy 3.

The surface morphologies (BSE images) of sealed and unsealed samples of three alloys corroded at 900 °C for 200 h are compared in Figure 6. On the surface of sealed corroded specimen Alloy 1, dispersive Al_2_O_3_ and nickel compound can be found as shown in Figure 6a. No stable intact protective oxidation layer existed on the alloy, and NaTaO_3_, NaCrS_2_, Na_2_O cannot be indicated directly as well. Figure 6b shows that, for the sealed corroded specimen of Alloy 2, a stable and continuous Al_2_O_3_ layer still kept and many small NiO diffused distributed on it. Some NaCrS_2_ also can be found in the Al_2_O_3_ layer on the surface. In addition to this, a few small cracks appeared in the outer layer. Figure 6c indicates that the structural integrity of protective Al_2_O_3_ layer was destroyed severely of Alloy 3, resulting that the internal of alloy exposed to the reaction environment. The surface products of unsealed prefabricated oxidation layer corroded specimen were similar to normal corrosion products. However, the ultimate integrity of the outer layer for Alloy 1 with prefabricated oxidation layer was better than normal Alloy 1(the normal experimental have been carried out before) as shown in Figure 6d [25,28]. There were no obvious cracks on the surface which can explain the improvement of hot corrosion resistance reflected in the kinetic curves in one respect. Complete and uniform oxides (thin NiO and internal Al_2_O_3_) layer formed on the Alloy 1 surface was remarkable. Although some small cracks appeared on the surface of corroded Alloy 2 as Figure 6e shown, there was no conspicuous spallation of protective layer, and primary Al_2_O_3_ and NiCr_2_O_4_ were maintained by and large which can prevent catastrophic hot corrosion to some extent. Figure 6f indicates that, for Alloy 3, the large cracks and a mass of Al_2_O_3_ spallation made the hot corrosion resistance reduce which reflected at the kinetic curves. NiCr_2_O_4_ spinel and Cr_2_O_3_ formed the main protective layer in the relatively complete area in corroded Alloy 3. In this area, the density of cracks and spallation was smaller than the area rich of Al_2_O_3_.

**Figure 7:**
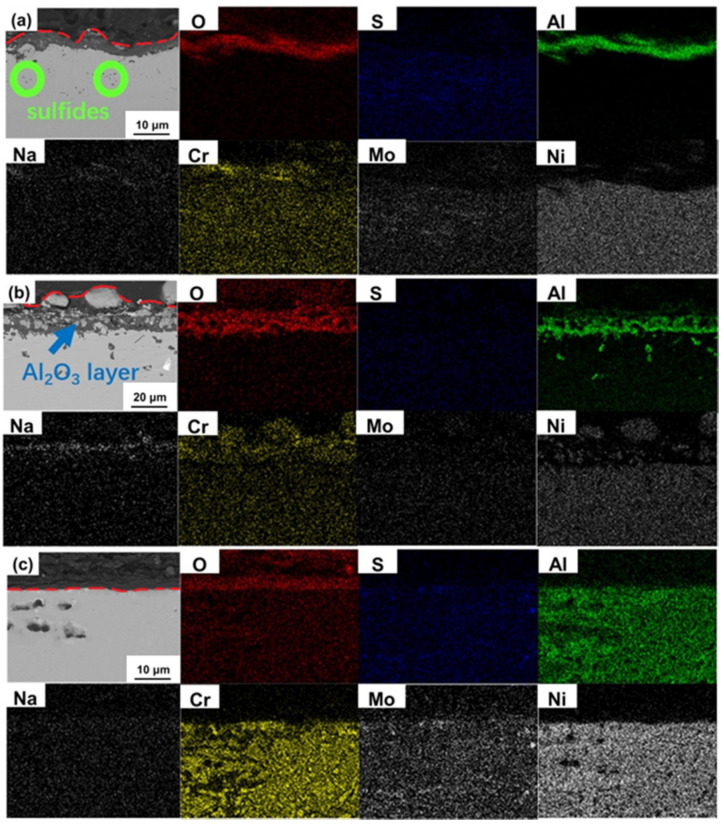
The cross-section morphologies and energy dispersive spectrometer (EDS) images of the sealed corroded samples at 900 °C for 200 h: (**a**) Alloy 1, (**b**) Alloy 2 and (**c**) Alloy 3.

**Figure 8:**
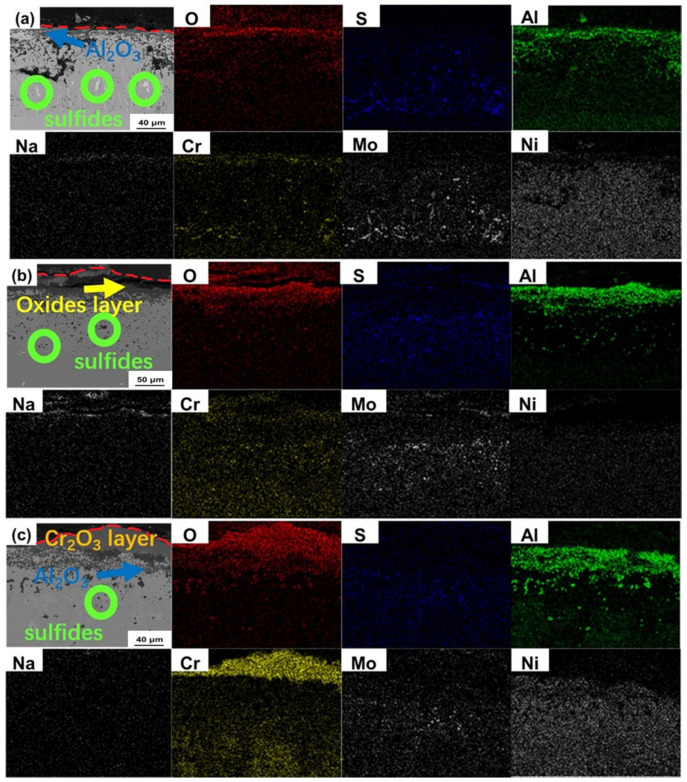
The cross-section morphologies and energy dispersive spectrometer (EDS) images of the unsealed corroded samples at 900 °C for 200 h: (**a**) Alloy 1, (**b**) Alloy 2 and (**c**) Alloy 3.

The cross-section morphologies and energy dispersive spectroscopy (EDS) images of the sealed corroded samples and unsealed corroded samples at 900 °C for 200 h with salt deposit are shown in Figure 7 and Figure 8, respectively. It can be seen that the outer Al_2_O_3_ layer kept complete after 200 h in sealed corrosion test for Alloy 1 as shown in Figure 7a. Although the Al_2_O_3_ layer was supposed to prevent the penetration of corrosive elements such as O^2−^ and S^2−^, some sulfides can still be found in the substrate combined with the EDS results. Figure 7b indicates that, for the sealed corroded specimen with prefabricated oxidation layer of Alloy 2, besides the Al_2_O_3_ layer can be indicated obviously, products containing sodium and chromium also existed in the outer layer. No apparent sulfides appeared under the oxide layer, but some rich aluminum phases can be found. There was no obvious protective oxide layer on the surface of sealed-corroded Alloy 3 sample as shown in Figure 7c. Even though no continuous and thick protective layer existed, the penetration of sulfur and oxygen was not serious. Not many oxides and sulfides can be found in the substrate, and only a few sulfides presented in the form of molybdenum sulfide. The outer layer of unsealed corroded specimen after 200 h of Alloy 1 was continuous but not dense Al_2_O_3_ layer with some NiO as shown in Figure 8a. It was worth noting that needle-like molybdenum sulfides and irregular chromium sulfides can be found in the inner diffusion layer. Although the interface flatness of the oxide layer and sulfide layer was still not very satisfying, the boundary of reaction zone and the substrate seemed smooth. Figure 8b indicates that, for the Alloy 2, the structure of the outer oxide layer after 200 h corroded with prefabricated oxidation layer was similar to high temperature oxidation experiment of nickel-based superalloy in atmospheric environment which consisted NiO, NiCr_2_O_4_, Al_2_O_3_ (denser than normal hot corrosion) and poor aluminum area [29,30]. Nevertheless, NaTaO_3_ detected in the surface of the specimen and existed inner sulfides meant the impact of melt-salt to the experiment. Meanwhile, sulfur and molybdenum were indicated mutually in the interior part of the diffusion layer near to the matrix. A discontinuous but thick Cr_2_O_3_ layer appeared on the surface of the unsealed corroded Alloy 3 which has prefabricated oxidation layer as shown in Figure 8c. Large diffused but not layered Al_2_O_3_ formed under the outer Cr_2_O_3_ or NiCr_2_O_4_ layer. Compared to Alloy 1 and Alloy 2, the strength of inner sulfides was so low. Interestingly, compared with CrS, MoS_2_ was more obvious in the EDS maps in spite of that Alloy 3 had the highest chromium content.

## 4. Discussion

### 4.1. The Influence of Oxidation Products on Hot Corrosion Properties

In a strict sense, pre-oxidation is a surface treatment process. The influence of this process on hot corrosion is mainly achieved by affecting the composition of surface components. It can be said more intuitively and clearly that the different composition and morphology of surface oxides will have a great impact on the hot corrosion of materials. Generally speaking, hot corrosion is regarded as a catastrophic degradation process because of its serious influence on the properties of alloy and its characteristic of becoming deteriorated with time extension [31]. The difficulty of forming stable and dense oxide layer is considered to play a decisive role in this process [32]. There is no doubt that different oxides have different effects on hot corrosion, and this effect is closely related to the structural stability.

Three main oxidation products (NiO, Al_2_O_3_ and Cr_2_O_3_) were obtained by pre-oxidation, respectively. As substances in direct contact with corrosive medium, it is necessary to first explore the possibility of the direct reactions with molten salt. The thermodynamic parameters of three oxides reacted with molten salt at 900 °C are listed in the Table 3 below. According to the standard Gibbs free energy and equilibrium constant, none of the three oxides can easily react with molten salt under normal condition directly. However, it can be clearly seen that the different oxides obtained from the pre-oxidation of different alloys will have a different impact on the hot corrosion properties of the alloys which was concluded from the above results that the hot corrosion properties of some alloys have changed greatly after the pre-oxidation. Through the experiment, it can be found that different surface oxidation products will bring different changes to the hot corrosion resistance of the alloy, or improve, or little impact. The effects of different oxides on the hot corrosion behavior of superalloys are described in detail below [33,34,35,36].

#### 4.1.1. The Effect of Cr_2_O_3_ on Hot Corrosion Properties

Cr_2_O_3_ is considered to be an excellent oxide to improve the hot corrosion resistance of alloys [37]. It is worth considering that no pure continuous oxide layer formed by Cr_2_O_3_ was found in the three pre-oxidized alloys. This is mainly due to the following factor: high volatility of Cr_2_O_3_ [38]. The vapor pressure of the oxide can measure the stability of the solid oxide at a certain temperature. If the vapor pressure of the oxide is high, the oxide is volatile. When the oxide evaporates, the free energy of the system changes as follow:

(1) $$\Delta G=RT\;ln\frac{p_{vapour}}{p_{vapour}^{\prime }}$$

$p_{vapour}$ is the actual pressure of oxide; $p_{vapour}^{\prime }$ is the vapor pressure at equilibrium.

The relation between vapor pressure and temperature can be derived from the Clapeyron formula:

(2) $$\frac{dp_{vapour}}{dT}=\;\frac{\Delta S^{\Theta }}{\Delta V}=\;\frac{\Delta H^{\Theta }}{T\left(\;V_{g}-V_{s}\right)}$$

V is the molar volume of the oxide; $\Delta S^{\Theta }$ is the standard entropy change of the oxide evaporation equilibrium reaction; $\Delta H^{\Theta }$ is the standard enthalpy change.

The molar volume of a solid $V_{s}$ is much smaller than the molar volume of a gas $V_{g}$, thus it can be negligible. At the same time, the vapor is regarded as ideal gas, meets the condition of $pV_{g}$ = RT, by substituting the upper equation and integrating it, and the result is as follow:

(3) $$lnp_{vapour}=\;-\frac{\Delta H^{\Theta }}{RT}+C$$

C is integral constant.

As can be seen from the above Equations (1)–(3), the higher the standard enthalpy of an oxide evaporates, the lower the vapor pressure, the more stable the oxide is. The standard enthalpy of evaporation of Al_2_O_3_ at 900 °C is higher than that of Cr_2_O_3_ after calculation. In addition to this, Cr_2_O_3_ can sublimate into CrO_3_ at about 900 °C. These explain why Al_2_O_3_ can form a protective layer but Cr_2_O_3_ cannot even in Alloy 3 with high Cr content as Figure 6c shown [38,39].

Although Cr_2_O_3_ did not appear in a stable layered structure in the prefabricated oxide layer, its effect on improving the hot corrosion resistance of the alloy was still very significant. For Alloy 1 and Alloy 2 that hot corrosion performance was greatly improved after pre-oxidation, a large number of oxides solid solution (α-Al_2_O_3_-Cr_2_O_3_, Ta_2_O_5_-CrO_3_) and spinel structure composite oxides (NiCr_2_O_4_) were found in their prefabricated oxide layers as shown in Figure 3. And these structures remained until the end of the hot corrosion experiment as shown in Figure 8. Other researches have shown, MoO_3_, Ta_2_O_5_ and NiO are easy to occur acid fluxing and lead to serious hot corrosion, while CrO_3_ is likely to produce mild basic fluxing [23]. This means that the factors leading to acidic fluxing are greatly reduced by the dissolution of oxides and solid phase reactions. This reduction can greatly improve the stability of the oxide layer and thus improve the hot corrosion properties of the alloys. In addition to reducing acid fluxing, Cr_2_O_3_ also has a great stabilizing effect on NiO. NiO, as a strong non-neutral oxide, greatly promotes the polarization of molten salt-alloy interface and aggravates the occurrence of hot corrosion [25]. The appearance of Cr_2_O_3_ can produce solid-phase reaction with nickel oxide as follow:

(4) $$NiO+Cr_{2}O_{3}=NiCr_{2}O_{4}$$

Compared with NiO, NiCr_2_O_4_ spinel structure oxide has a compact structure and electrochemistry neutrality. These characteristics can effectively improve the hot corrosion properties of alloys. This improvement was particularly evident in Alloy 1 which contained a large amount of NiO in the oxide layer as shown in Figure 4a. For alloy 2, whose oxide layer was stabilized by itself, and alloy 3, whose surface oxide layer was Al_2_O_3_, the improvement effect is not obvious. At the same time, the results of the tube sealing experiments also showed that Cr_2_O_3_ did indeed evaporate during the reaction (difficult to react directly with the melting salt) as shown in Figure 7.

Of course, all the above-mentioned discussions are obtained by pre-oxidation in air. If the alloy is under the cover of molten salt for a long time, because of the solid-liquid interface between the alloy and molten salt, there is no obvious volatilization, so stable Cr_2_O_3_ can still be formed on the surface of the alloy, and provide excellent hot corrosion resistance for the alloy such as Alloy 3 [23,40]. In general, Cr_2_O_3_ in the form of solid solution or non-volatile Cr_2_O_3_ has a good hot corrosion resistance improvement. So, the pre-oxidation layer (Cr_2_O_3_ layer) of alloy with high Cr content has no significant improvement effect compared to no pre-oxidation layer.

#### 4.1.2. The Effect of NiO on Hot Corrosion Properties

The role of NiO is also an important point in the analysis of oxides affecting hot corrosion. Although few researchers believe that NiO has a positive effect on hot corrosion, it must be admitted that NiO has a useful side in hot corrosion to some extent. In the three kinds of pre-oxidized alloys, NiO exists more or less in the prefabricated oxidation layer as the single phase because of the oxidation characteristics of nickel-based superalloys. However, after a long period of hot corrosion it did not survive effectively, either in the air or in tube sealing experiments as shown in Figure 7 and Figure 8. It is no doubt that pure NiO does not play a very good role in improving the hot corrosion properties of the alloys. However, NiO, as an oxide directly contacting with melt salt at the beginning, can prevent the melt salt from contacting with the matrix quickly to a certain extent, which provides the possibility for the formation of other stable oxides, which is also the reason why the hot corrosion resistance of Alloy 1 was improved to a certain extent after pre-oxidation. At the same time, however, the presence of NiO in the form of composite oxides and some NiO as the “matrix” of other oxides can still be found. It explains the role of NiO-stable dispersion phase and bulk spinel phase as the NiCr_2_O_4_ mentioned above [32]. This stabilizing effect is especially important when the alloy subjected to thermal shock. In addition, NiO can reduce the volatilization of Cr_2_O_3_ by forming a compound oxide with Cr_2_O_3_ [41].

#### 4.1.3. The Effect of Al_2_O_3_ on Hot Corrosion Properties

The effect of Al_2_O_3_ on improving the hot corrosion performance of the alloy has been showed incisively and vividly in the experiment. After 200 h of tube sealing test, the Al_2_O_3_ in the alloy prefabricated oxide layer was very intact in Alloy 1 and 2 while other oxides (notably NiO) were depleted basically. It indicates that Al_2_O_3_ has good chemical stability under the condition of 900 °C molten salt coating. Considering the Al_2_O_3_ itself has such advantages as compactness and excellent electrical neutrality, the factors that improve the hot corrosion performance of the alloy are very comprehensive by Al_2_O_3_ [42,43].

However, the performance of Al_2_O_3_ in atmospheric hot corrosion is closely related to its morphology and distribution. It can be seen that after hot corrosion, there was still a dense and complete Al_2_O_3_ layer on the surface of Alloy 1 and 2 which had a significantly promotion in hot corrosion resistance after pre-oxidation. The appearance of this layer of Al_2_O_3_ ensured the densification of the oxide layer and reduced the possibility of contact between molten salt and alloy. Further combining with the inspection of the kinetic curves of the hot corrosion (Figure 4) can obtained that although basic fluxing still occurred, alloy-induces acidic fluxing (Equations (5)–(7)) was inhibited greatly by preventing the direct contact between the molten salt and the matrix and reducing the oxidation of the elements (such as Mo and W) inside the alloy [44]:

(5) $$Mo+\;\frac{3}{2}O_{2}=MoO_{3}$$

(6) $$MoO_{3}+Na_{2}SO_{4}=Na_{2}MoO_{4}+SO_{3}$$

(7) $$M_{x}O_{y}+\;yMoO_{3}\;\left(in\;Na_{2}SO_{4}\right)=xM^{\frac{2y}{x}+}+\;yMoO_{4}^{2-}$$

$M_{x}O_{y}$ means neutral oxides such as NiO, Al_2_O_3_ and Cr_2_O_3_.

From this point of view, a layer of stable and compact Al_2_O_3_ plays a positive role in improving the hot corrosion properties of the alloy. At the same time, some dispersed Al_2_O_3_ can be found beneath the stable oxide layer as shown in Figure 3e. And in general, dense Al_2_O_3_ layer is difficult to form, this kind of dispersed loosened Al_2_O_3_ is more likely to form as displayed in Alloy 3 that based on the acid-base melting model (Equations (8)–(11)):

(8) $$Al_{2}O_{3}+3SO_{3}\;\to Al_{2}{\left(SO_{4}\right)}_{3}$$

(9) $$Al_{2}{\left(SO_{4}\right)}_{3}\;\to 2Al^{3+}+3SO_{4}^{2-}$$

(10) $$SO_{4}^{2-}\;\to \;SO_{3}+O^{2-}$$

or:

(11) $$Al_{2}O_{3}+\;O^{2-}\;\to 2AlO_{2}^{-}$$

When the above reactions occur at the beginning of hot corrosion without pre-oxidation or in low oxygen environment, Al_2_O_3_ on the surface of the alloy will be continuously consumed and precipitated into loose Al_2_O_3_ at the gas-liquid interface which leads to the loss of protective oxide layer, forcing the contact of alloy matrix with molten salt as shown by normal hot corrosion. And these loose Al_2_O_3_ will easily become the origin of cracks and expansion channels, molten salt diffusion channels and then break off the integrity of the alloy. To sum up, the effect of Al_2_O_3_ on the hot corrosion performance of the alloy is largely determined by the structure of Al_2_O_3_ itself. Although the stable and dense Al_2_O_3_ layer may be consumed due to hot corrosion, the dissipation effect is not obvious along with the oxidation in hot corrosion, and the protection of the oxide layer is guaranteed. However, when severe hot corrosion occurs, dispersed Al_2_O_3_ will not only have no protective effect, but also accelerate the occurrence of hot corrosion and spallation [45,46,47].

#### 4.1.4. The Hot Corrosion of the Surface Oxide Layers Composed of Multiple Oxides

**Figure 9:**
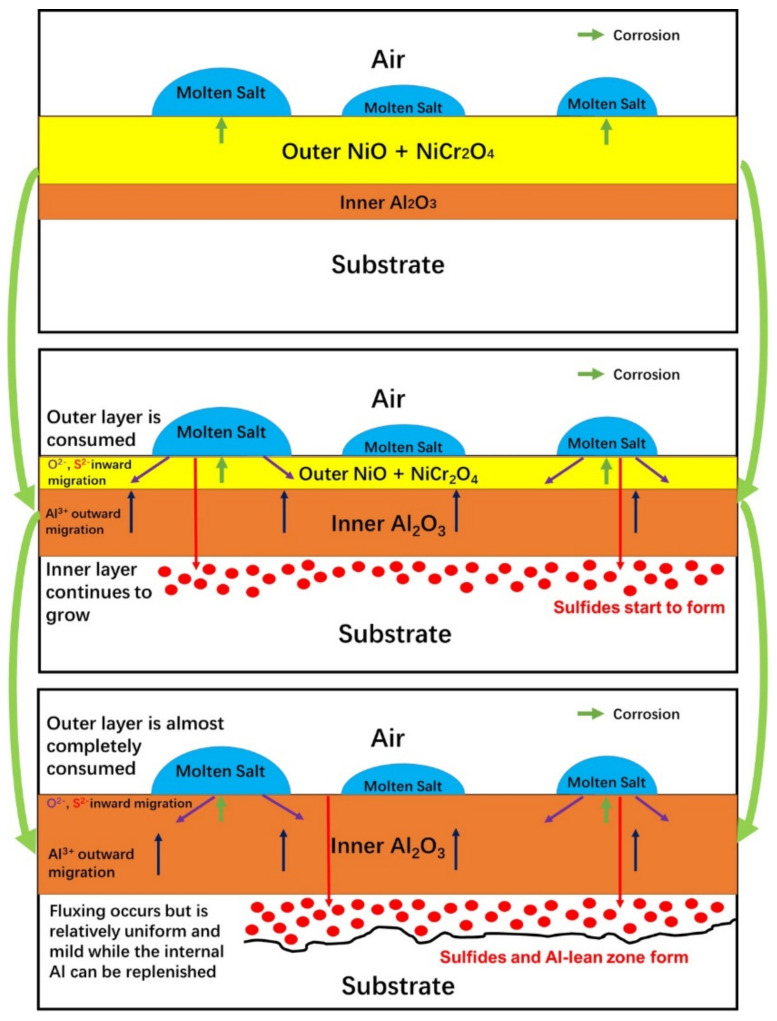
The schematic diagram of the oxides’ changes in hot corrosion after pre-oxidation.

The functions of different oxides after pre-oxidation are described above separately. It should also be considered there is a relationship between different oxides in the process of hot corrosion. The schematic diagram of the oxides’ changes in hot corrosion after pre-oxidation is shown as Figure 9. It can be found that after the prefabricated oxidation layer, the surface oxides after hot corrosion are more complete than those not pre-oxidation, which is related to the fact that Na_2_SO_4_ is consumed by the surface oxides to a certain extent, and new oxides can be added in the internal matrix to ensure the integrity of the oxidation layer. Specifically, NiO is consumed on the surface, but the more stable Al_2_O_3_ and Cr_2_O_3_ are formed on the inside, just as Alloy 1 and 2 behaved in pre-oxidized hot corrosion. The reaction is not severe enough to make the oxide layer lose its protective effect which can be reflected in Figure 4. So, in summary, prefabricated a stable and dense oxide layer can significantly improve the hot corrosion resistance of the alloys especially the Al_2_O_3_ layer [11,24,48].

The role of NiO, Al_2_O_3_ and Cr_2_O_3_ in hot corrosion on the surface layer can be summarized above. Oxidation, as an extremely complex and multicomponent reaction, produces a variety of products. The dominant role of major surface oxides products on hot corrosion performance can be clearly obtained. In the above paragraph, the possibility of the most ideal pre-oxidation to improve the hot corrosion resistance of single crystal alloy is described. It is based on the formation of a stable surface Al_2_O_3_ layer, as shown by the Alloy 2. If the stable Al_2_O_3_ layer is not obtained by pre-oxidation, such as the surface oxide is mainly NiO (high Ni content), the improvement of hot corrosion resistance is limited which is reflected in the fact that, in the initial stage of hot corrosion, the dense NiO reduces the speed of the melting process, but as the reaction progresses, the dense NiO is exhausted and other stable oxides layers are not formed (NiO to some extent slows the internal diffusion of oxygen), catastrophic hot corrosion will eventually occur as shown by the Alloy 1. When the Cr content is extremely high, Cr_2_O_3_ forms on the surface. Due to its rapid formation and volatilization, the oxide layer dominated by Cr_2_O_3_ is not thick, and the formation of NiO and Al_2_O_3_ is affected to some extent. Therefore, when hot corrosion occurs, its effect of improving hot corrosion resistance is not obvious, but due to its own stability, catastrophic hot corrosion does not occur as displayed in the Alloy 3.

### 4.2. The Formation and Distribution of Sulfides in the Hot Corrosion Test

**Figure 10:**
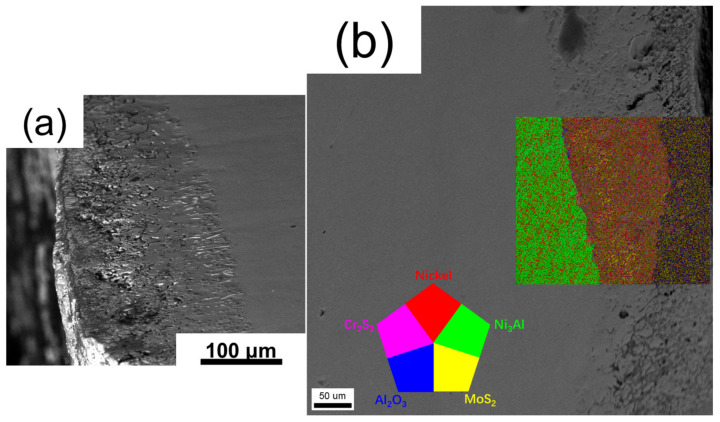
The possible distribution of different phases of Alloy 1 (4Cr) after 200 h hot corrosion at 900 °C by EBSD. (**a**) The cross-section morphology (SE image) of the sample and (**b**) The distribution of different phases by EBSD.

The role of oxides in hot corrosion is described in detail above. Although sulfides are important products of hot corrosion, the research on them is not very thorough, due to the high reaction complexity and easily be influenced by many factors. Nevertheless, a careful analysis of the sulfides will help us to analyze the process of hot corrosion. Through the observation of the cross-section structure of the three alloys subjected to atmospheric hot corrosion after pre-oxidation, it can be found that the sulfides content of the alloys decreased with the increase of Cr content (the reduction of the severity of corrosion reaction). This can be simply attributed to the difference in the reaction degree of Na_2_SO_4_. The more severe the hot corrosion reaction is, the greater the role of Na_2_SO_4_ is reflected, and the large number of S elements also proves the point [49]. Further, although sulfides may correspond to the degree of reaction, the composition of sulfides after hot corrosion of alloys of different compositions is also different [50]. In view of the low content of sulfides and its existence mainly under the oxide layer of the alloy, EBSD method was used to conduct a detailed study on the sulfides after hot corrosion of the alloy. Figure 10 shows the distribution of different phases of Alloy 1 (4Cr) after 200 h hot corrosion at 900 °C by EBSD. First of all, there is no doubt from the figure that the alloy appeared obvious stratification after hot corrosion. From the outside to the inside were oxide layer, Al-lean layer and matrix. Al_2_O_3_, the main product of the outer oxide layer, and Ni_3_Al and Ni-base solid solution of the inner matrix can be clearly observed as normal. According to EBSD analysis of the cross-section, the area in the middle is the main accumulation area of sulfides. In which, the existence of MoS_2_ can be found obviously as the yellow part in the Figure 10.

**Figure 11:**
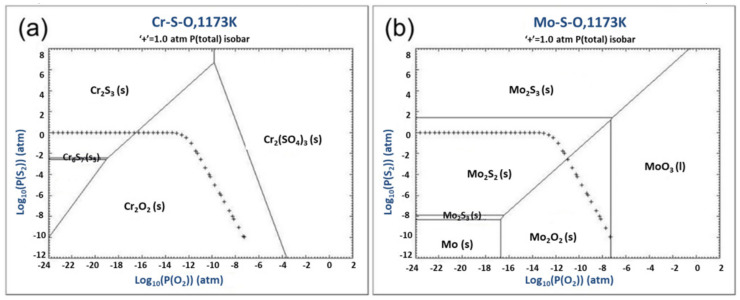
The schematic diagram of the formation of sulfides and oxides under different gas partial pressures [54]. (**a**) Cr-S-O ternary phase composition. and (**b**) Mo-S-O ternary phase composition.

Combined with the previous results and the schematic diagram of the formation of sulfides and oxides under different gas partial pressures as shown in Figure 11, the presence of sulfides accords with the following characteristics. First of all, chromium sulfide (Cr_2_S_3_) and molybdenum sulfide (MoS_2_) are the main sulfide products of the alloy after hot corrosion which depends on the partial pressure of oxygen and the partial pressure of sulfur. Secondly, MoS_2_ is more distributed in long strips, while Cr_2_S_3_ is distributed in spots as shown in Figure 11. Finally, MoS_2_ appears more obviously when the Cr content is less or the Cr consumption is more serious. According to results of the EDS, the combination of Cr, Mo and S may not be compounds with certain atomic proportions. However, it can still be showed that Cr_2_S_3_ is the most preferred form of sulfide, while the appearance of MoS_2_ indicates that the hot corrosion of the alloy has been very severe. The presence and representation of the sulfide has been carefully analyzed, which means that the degree of hot corrosion of the alloy can be determined by the sulfide, since the outer oxides undoubtedly has the possibility of separation and spalling while sulfides are not easily shed because they lie on the inside [51,52,53].

## 5. Conclusions

The effect of different surface native pre-oxides on hot corrosion of single crystal nickel-based superalloy at 900 °C is studied. Pre-oxidation and tube sealing experiments are used to determine the role of oxides on hot corrosion in term of reaction. Then, the sulfides produced in hot corrosion is further described to relate the surface oxides. The main conclusions are as follows:

1. The thin oxide layer mainly composed of Cr_2_O_3_ forms on the surface of the pre-oxidized alloy with high Cr content (20%). When the Cr content is less than 10%, the outer oxide layer of the pre-oxidized alloy is dominated by NiO and Al_2_O_3_ multilayer structure. When the Ni content (up to 65%) is high, continuous Al_2_O_3_ layer is difficult to form.
2. Cr_2_O_3_ is an oxide with strong hot corrosion stability, but it is difficult to maintain a large scale of oxide layer by pre-oxidation because of the high volatility, so the effect of pre-oxidation is not obvious.
3. NiO is easily consumed in hot corrosion, but it can form a stable NiCr_2_O_4_ with Cr_2_O_3_ (reduce the volatilization of Cr_2_O_3_), and the pre-oxidized NiO can consume some molten salt and promote the formation of a dense Al_2_O_3_ layer during this consumption process.
4. A stable, dense Al_2_O_3_ layer with a large scale, which can form by pre-oxidation, is useful for improving the hot corrosion resistance by preventing the reaction between the alloy and the molten salt.
5. A multilayer structure pre-oxide layer containing an external NiO + NiCr_2_O_4_ and an internal Al_2_O_3_ can effectively improve the hot corrosion resistance of the alloy, mainly through the consumption of salt by NiO and the formation of a stable and dense Al_2_O_3_ layer at the beginning of the reaction.
6. The generation of sulfides in hot corrosion is often accompanied by the co-occurrence of Cr, Mo and S. Generally, the generation of Cr-S compounds has a higher priority, while the formation of Mo-S compounds in a large amount means the hot corrosion is carried out to a catastrophic state. At the same time, the formation of oxides and sulfides is controlled by the different partial pressure of the gas.

## Figures and Tables

**Table 1:** Composition (wt.%) of alloys used in the experiments.

Alloy	Cr	Co	W	Mo	Al	Ta	Re	Hf	Ni
Alloy 1	4.0	7.5	4.0	2.5	6.5	7.0	3.0	0.1	Bal.
Alloy 2	7.0	7.5	4.0	2.5	6.5	7.0	3.0	0.1	Bal.
Alloy 3	20.0	7.5	4.0	2.5	6.5	7.0	3.0	0.1	Bal.

**Table 2:** The atomic percentages (at.%) of elements at different locations measured by EDS in Figure 3.

Element	1	2	3	4	5
O	50.81	52.79	59.10	60.00	41.95
Al		24.90	39.39	33.73	26.27
Cr		5.18	0.67	2.14	9.23
Co	4.51	1.68		1.35	2.06
Ni	44.68	13.73	0.84	2.78	17.90
Others		1.72			2.58

**Table 3:** The thermodynamic parameters of three oxides reacted with molten salt at 900 °C.

Reaction	ΔG (kcal)	ΔG (kJ)	Log (K)
$\mathrm{NiO}+{\mathrm{Na}}_{2}{\mathrm{SO}}_{4}={\mathrm{Na}}_{2}{\mathrm{NiO}}_{2}+{\mathrm{SO}}_{3}\;\left(g\right)$	95.098	398.069	−17.72
${\mathrm{Al}}_{2}O_{3}+{\mathrm{Na}}_{2}{\mathrm{SO}}_{4}=2{\mathrm{NaAlO}}_{2}+{\mathrm{SO}}_{3}\;\left(g\right)$	47.923	200.511	−8.929
${\mathrm{Cr}}_{2}O_{3}+{\mathrm{Na}}_{2}{\mathrm{SO}}_{4}={\mathrm{Na}}_{2}{\mathrm{Cr}}_{2}O_{4}+{\mathrm{SO}}_{3}\;\left(g\right)$	73.318	429.289	−13.66
